# Measurement of Carbon Emission Transfer in China's Construction Industry and Analysis of Spatial and Temporal Distribution of Carbon Emissions

**DOI:** 10.1002/gch2.202400368

**Published:** 2025-02-13

**Authors:** Wenwen Xiao, Wenhao Song, Xuemei Pei, Lili Wang

**Affiliations:** ^1^ School of Management Engineering Shandong Jianzhu University Jinan 250101 China; ^2^ School of Business Shandong Jianzhu University Jinan 250101 China

**Keywords:** carbon emissions from construction, carbon transfers, complex networks

## Abstract

The construction industry is an important material production sector in China's national economy, and the trade of goods and services between regions may lead to the transfer of carbon emissions from the construction industry. This study constructs a multiregion input–output table model containing 27 industries in 30 provinces in China from 2007 to 2017. It measures and analyzes carbon emissions and carbon transfer in China's construction industry, constructs a carbon emission transfer network for China's construction industry by combining a complex network model, and analyzes the spatial and temporal transfer structural characteristics of its network indicators. The results show that most provinces with higher carbon emissions from the construction industry are concentrated in the eastern coastal areas and regional center provinces, and in addition to the frequent carbon transfers within economically developed regions, the resource‐intensive provinces also frequently have carbon transfers with economically developed provinces. Based on the results of this study, a differentiated carbon emission reduction plan is formulated, and policy recommendations for optimizing carbon emission reduction in the construction industry in each region are proposed.

## Graphical abstract

The carbon emission problem of the construction industry poses a challenge to the global environment. This study analyzes the role of Chinese provinces in influencing carbon emissions by calculating indicators based on the establishment of a complex network by integrating data into a multiregional input–output model through the measurement of carbon emissions and carbon transfers.

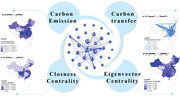

## 1 Introduction

Since the beginning of the 21st century, China's carbon emissions have increased annually. Since 2007, China has surpassed the United States as the world's top emitter of carbon dioxide in terms of total carbon emissions. To cope with the increasingly severe environmental problems, President Xi Jinping proposed the dual‐carbon targets of “carbon peaking” by 2030 and “carbon neutrality” by 2060 at the 75th session of the UN General Assembly. How can economic growth targets be effectively balanced? Achieving green and low‐carbon development and reaching the “dual‐carbon” goal on schedule while effectively considering the goal of economic growth is challenging and a problem to be solved. At the end of the 2023, the China Association of Building Energy Efficiency released the “Research Report on Carbon Emissions from China's Buildings and Urban Infrastructures,” which indicated that the total amount of carbon emissions from the construction industry in the country was ≈1.5 million tons, which is the highest in China. The total carbon emissions from the entire construction industry were 5.01 billion tons of CO_2_, accounting for 47.1% of national energy‐related carbon emissions. In addition, the country is focusing on strengthening domestic consumer demand and interregional trade to address its over‐reliance on external demand. According to the data released by the Ministry of Commerce, China's domestic trade exceeded 10 trillion CNY for in 2017, accounting for ≈13% of GDP, second only to the manufacturing industry, and significantly contributing to economic development, employment expansion, and tax revenue growth. Therefore, controlling the carbon emissions of the construction industry and clarifying the amount of carbon emissions transfer caused by domestic interregional trade in the construction industry is crucial to achieving China's energy conservation, emission reduction, and dual‐carbon targets, and is of great significance for realizing the low‐carbon development of the construction industry.

Based on this background, scholars have begun to research carbon emission reduction in the construction industry. Jia et al.^[^ ^1^, ^2^ ^]^ indicated that China's carbon dioxide emissions significantly impact global climate change. Yang et al.^[^ ^3^ ^]^ constructed a comprehensive evaluation index system and established a coupled coordinated development index model, state space model, and crisis transformation degree model to conduct a crisis transformation study on the coupled effects of the carbon emission reduction‐environmental protection‐economic development system of China's buildings from 2010 to 2020. Su et al.^[^ ^4^ ^]^ constructed an economic and carbon emission correlation model between provincial industries using the input–output method in China's multiregion input–output (MRIO) model. They analyzed industrial sectors with a better national carbon emission correlation. Through the industrial linkage analysis method, trends in the influence and inductance coefficients of the industrial sectors' economy and carbon emissions were investigated, and the critical provincial carbon emission reduction sectors in China were identified from the supply and consumption sides. Ding et al.^[^ ^5^ ^]^ constructed a multi‐industry dynamic stochastic general equilibrium model that incorporated environmental externalities and carbon abatement policies to investigate the dynamic effects of industry technology shocks, tax policies, and abatement policy adjustment shocks on the macroeconomy, environment, and industry emissions under two policy scenarios: permit trading and carbon tax. In addition, interregional close trade flows drive the flow of products and services, which will transfer a large amount of carbon dioxide emissions.^[^ ^6^ ^]^ Xi et al.^[^ ^7^ ^]^ constructed a regional carbon neutrality assessment framework that included embodied carbon transfers and carbon sequestration service flows. The framework was then applied to assess carbon neutrality and its influencing factors in Chinese provinces, showing that interregional carbon transfer is an important factor influencing carbon emissions. Yong et al.^[^ ^8^ ^]^ used a comprehensive approach combining social network analysis and a coupled coordination model to measure the carbon neutrality of 67 countries along the Belt and Road from 2010 to 2016 and estimated the carbon transfer network of the FDI network and the carbon transfer network of 67 countries along the Belt and Road from 2010 to 2016. Han et al.^[^ ^9^ ^]^ measured the carbon transfers in, carbon transfers out, and net carbon transfers of each province based on the MRIO model and the input–output data of 30 Chinese provinces, and built the model based on a model to provide policy recommendations in line with the actual situation of each region, which confirmed the necessity of subregional research. Fang et al.^[^ ^10^ ^]^ constructed a complex interdependent network structure based on the 2016 world MRIO table and interindustry production factor transfer. Through input–output and complex network analyses, the individual nodes and structural features of the carbon flow network were explored, and the carbon transfer between countries was revealed from a multiregional perspective. Consequently, the transfer of carbon dioxide from trade flows has become an increasingly important issue that can significantly affect the effectiveness of carbon emission reduction measures.^[^ ^11^ ^]^ Wu et al.^[^ ^12^ ^]^ examined the decoupling relationship between economic output and carbon emissions by focusing on China's construction industry, which is a pillar industry for national economic growth and contributes a large amount of carbon emissions. Yang et al.^[^ ^13^ ^]^ investigated the impact of digital city construction on carbon emissions in China and its transmission mechanism using panel regression and mediating effect models. Nasir et al.^[^ ^14^ ^]^ indicate that green and sustainable supply chain management practices have been developed to reduce the negative consequences of environmental production and consumption processes. Xiao et al.^[^ ^15^ ^]^ developed a comprehensive analytical framework to explore the impact of new urbanization on carbon emissions from urban buildings in three dimensions: scale, average, and structure. Yuan et al.^[^ ^16^ ^]^ indicated that clarifying the historical characteristics and factors influencing carbon emissions in the construction industry is essential for formulating targeted emission reduction policies. Zhou et al.^[^ ^17^ ^]^ indicated that the construction industry is one of the most energy‐intensive industries in China, contributing to high carbon emissions and hindering efforts to reduce emissions. Li et al.^[^ ^18^ ^][^ ^19^ ^][^ ^20^ ^]^ indicated that the construction industry emits 36% of global GHG emissions in all sectors. China is the world's largest carbon emitter. Liu et al.^[^ ^21^ ^]^ indicated that it is a significant source of global energy consumption and GHG emissions, anticipating that its developmental trends are crucial for energy conservation and emission reduction. Jackson et al.^[^ ^22^ ^][^ ^23^ ^][^ ^24^ ^]^ indicated that the construction industry is facing increasing pressure to reduce carbon emissions. An important first step is to quantify emissions from construction projects, enabling designs to be changed and reducing emissions. Wu et al.^[^ ^25^ ^]^ evaluated the carbon emissions of this industry from a life‐cycle perspective, including extraction, manufacturing, construction, and construction‐related transportation. Li et al.^[^ ^26^ ^]^ developed an innovative C‐shaped strategic map to address the CER barriers in the construction industry by integrating the dual dimensions of time and space. Luo et al.^[^ ^27^ ^]^ analyzed the direct and indirect impacts of population growth, economic development, and industrial restructuring on carbon emissions and proposed targeted policy recommendations, including sustainable urban planning and green building design. The construction sector is a key factor in the fight against climate change. A clear and comprehensive account of its carbon emission responsibilities forms the basis for effective emission‐mitigation actions. The intersectoral and interregional trade in the construction sector complicates emission responsibility allocation.^[^ ^28^ ^]^ In addition, interregional trade brings economic and carbon emission spillover and feedback effects.^[^ ^29^ ^]^

In conclusion, the issue of carbon emissions transfer in the construction industry has attracted extensive attention worldwide, including from domestic and foreign government agencies, international organizations, and scholars. At present, research on carbon emission measurement methodologies and carbon emission policy recommendations for the construction industry is relatively mature and fruitful, and these studies have provided theoretical and policy recommendations for solving the global carbon emission problem. However, current research on carbon emissions from the construction industry, whether at the national or provincial level, focuses on its own economic, technological, and spatial effects, as well as other aspects of its impact. However, as China's domestic trade becomes increasingly closer, some scholars have begun to pay attention to the interregional trade carbon emission transfer and found that the trade carbon emission transfer and the role of each province in carbon transfer have a significant impact on the carbon emission reduction results, interregional trade exists in the economy with spillover and feedback effects, and in carbon emissions. However, studies exploring the impact of each province on carbon emissions in the construction industry from the perspective of interregional carbon emissions transfer are lacking. In addition, most existing emission transfer studies are based on foreign trade or a single region as the research object; there are few theoretical and empirical studies related to domestic trade and multiple regions.

Therefore, based on existing research, combined with the actual situation of carbon emission in the construction industry, we calculated the carbon emission transfer in the construction industry based on the MRIO table and the energy table, and preliminarily analyzed the characteristics of the distribution of carbon emission transfer in China's construction industry and the transfer situation. Then, complex network theory was used to construct the national construction industry carbon emission transfer network, clarify the position of each province, and empirically analyze the indicators. Finally, we propose targeted improvement measures and suggestions.

Based on this, this study attempts to contribute the following: first, it calculates the amount of interregional construction carbon emission transfer in China based on the MRIO model, and constructs a national construction carbon emission transfer network using complex network theory to clarify the position of each province in the construction carbon emission transfer network. Second, we analyzed each province's overall and individual indicators in the national construction industry's carbon emission transfer network by combining input–output theory and complex network theory to provide effective carbon reduction policies for the construction industry from the perspective of interregional carbon transfer in the construction sector.

## 2 Research Methods

### 2.1 Modeling of Interregional Carbon Transfer in the Construction Industry

#### 2.1.1 Carbon Emission Measurement Modeling in the Construction Industry

Considering the strong correlation between the construction and other industries, carbon emissions from the construction industry can be divided into energy and nonenergy substance carbon emissions. Energy carbon emissions are generated by the primary energy directly consumed by the construction industry, and nonenergy material carbon emissions are closely related to those of other upstream and downstream enterprises, which are expressed as carbon dioxide released during the production and transport of construction materials. Drawing on Jin et al.,^[^ ^30^ ^]^ considering data availability, the direct carbon emission sources were identified as three primary (coal, crude oil, and natural gas) and five secondary (coke, gasoline, paraffin, diesel oil, and fuel oil) and eight other energy sources involved in the life‐cycle of the building, and the discounted standard coal and carbon emission coefficients of each energy source are shown in **Table** **1**; indirect carbon emission sources are identified as primary energy sources in the life cycle of the building. The indirect carbon emission source was identified as the consumption of five primary construction materials, namely glass, wood, cement, aluminum, and steel, which are mainly used in the construction process. The carbon emission and recovery coefficients of each construction material are shown in **Table** **2**.

**Table 1:** Carbon emission factors for different energy types.

Energy	Coal	Crude oil	Petroleum	Coke	Petrol	Diesel	Diesel oil	Fuel oil
Carbon emission factors	2.69 Kg CO_2_ Kg^−1^	2.76 Kg CO_2_ L^−1^	2.09 Kg CO_2_ m^−^^3^	3.14 Kg CO_2_ Kg^−1^	3.14 Kg CO_2_ L^−1^	2.56 Kg CO_2_ L^−1^	2.73 Kg CO_2_ L^−1^	3.14 Kg CO_2_ L^−1^

**Table 2:** Carbon emissions of primary building materials, recovery factors.

Building material	Clinker	Nylon	Steels	Aluminum	Lumber
Carbon emission factors	0.815 kg kg^−1^	0.9655 kg kg^−1^	1.789 kg kg^−1^	2.6 kg kg^−1^	−842.8 kg m^−^^3^
Recovery factor	—	0.7	0.8	0.85	0.2

In summary, using the IPCC coefficient method, this study measured the carbon emissions from buildings in 30 Chinese provinces from 2007 to 2017 (the carbon mentioned and measured in this study is CO_2_). The carbon‐emissions calculation model for the construction industry is as follows

(1) $$C=c_{1}+c_{2=}\sum_{i=1}^{8}M_{i}\times f_{i}+\sum_{j=1}^{5}N_{j}\times q_{j}\times \left(1-\alpha_{j}\right)$$

where *c*_1_ indicates carbon emissions from energy substances, *c*_2_indicates nonenergy material carbon emissions, *M*_*i*_indicates consumption of energy type *i*, *f*_*i*_indicates emission factor for energy source *i*, *N_j_* indicates consumption of construction material type *j*, *q_j_* indicates emission factor for construction material type *j*, and α_*j*_ indicates its recovery factor (carbon emissions measured in tons, subsequent references to carbon emissions and transfers are also in tons).

#### 2.1.2 Construction of a Model for Measuring Interregional Carbon Emission Transfer in the Construction Industry

Carbon transfer refers to the process by which industries or activities emitting carbon move from one country to another.^[^ ^31^ ^]^ This transfer is typically accompanied by the acceleration of international trade, industrial division of labor, and globalization. Currently, there are two methods for measuring the interregional transfer of carbon emissions: the life‐cycle^[^ ^32^ ^]^ and input–output approaches.^[^ ^33^ ^]^ The input–output method is subdivided into single‐region and MRIO methods. This study uses the MRIO method to measure the transfer of carbon emissions from the construction industry among 30 Chinese provinces, using China's noncompetitive input–output model (total output excluding imports).

According to the principle of the MRIO model, the specific steps for measuring the carbon emission transfer of the construction industry among the provinces are as follows:

In the input–output method, the following relationship exists between the total output and final consumption

(2) $$X={\left(I-A\right)}^{-1}\;Y$$

where *X* represents the column vector of the total output of the construction sector, *Y* represents the column vector of final use, *A* represents the matrix of direct consumption coefficients, and *I* represents the matrix of units whose order is harmonized with the order of the matrix of direct consumption coefficients. (*I* − *A*)^− 1^represents the Leontief inverse matrix, which represents the additional output of one sector necessary to satisfy the final demand per unit in another sector.

In the MRIO analysis, the carbon emission factor for the construction sector is calculated as follows

(3) $$\theta^{r}=\frac{Q^{r}}{X^{r}}$$

where θ^*r*^ denotes the direct carbon emission factor for the construction sector in region *r*, *Q^r^* denotes the carbon emissions from the construction sector in region *r*, and *X^r^* denotes the total output of the construction sector in region *r*. Combined with the Leontief inverse matrix, the full carbon emission factor for the construction sector is obtained as

(4) $$\varepsilon =\theta \times {\left(I-A\right)}^{-1}$$

Take Region *r* for example

(5) $$\begin{array}{cc} \varepsilon^{r} & =\left[\begin{array}{ccccccc} 0 & & & & & & \\ & \ddots & & & & & \\ & & 0 & & & & \\ & & & \theta^{r} & & & \\ & & & & 0 & & \\ & & & & & \ddots & \\ & & & & & & 0 \end{array}\right]\\ & \;\times {\left[1-\left(\begin{array}{ccccccc} a_{11}^{rs} & a_{12}^{rs} & a_{13}^{rs} & \cdots & \cdots & \cdots & a_{1n}^{rs}\\ a_{21}^{rs} & a_{22}^{rs} & a_{23}^{rs} & \cdots & \cdots & \cdots & a_{2n}^{rs}\\ \cdots & \cdots & \ddots & \cdots & \cdots & \cdots & \cdots \\ \cdots & \cdots & \cdots & \ddots & \cdots & \cdots & \cdots \\ \cdots & \cdots & \cdots & \cdots & \ddots & \cdots & \cdots \\ \cdots & \cdots & \cdots & \cdots & \cdots & \ddots & \cdots \\ a_{n1}^{rs} & a_{n2}^{rs} & a_{n3}^{rs} & \cdots & \cdots & \cdots & a_{nn}^{rs} \end{array}\right)\right]}^{-1} \end{array}$$

where θ^*r*^ is the carbon emission factor for the construction sector in region *r*. Because we investigated the amount of carbon transferred from the construction sector between regions, the carbon emission factor for sectors other than the θ^*r*^ construction sector (row 24) is set to 0; the subscripts of the matrix of direct consumption coefficients indicate that there are sectors from 1 to *n* (= 1,2,3,.,27)

We multiplied the total carbon emission coefficient of the construction sector by the end‐use component to obtain the total carbon emissions from the construction sector, which is the amount of carbon transfer caused by the end use in region *s* to the construction sector in region *r*. Therefore, we can measure the interregional carbon transfer from the construction sector in terms of total carbon emissions; that is, interregional carbon transfer from the construction sector is

(6) $$O^{rs}=\left[\begin{array}{ccccccc} 0 & 0 & \cdots & \cdots & \cdots & \cdots & 0\\ \cdots & \ddots & \cdots & \cdots & \cdots & \cdots & \cdots \\ \cdots & \cdots & \ddots & \cdots & \cdots & \cdots & \cdots \\ \varepsilon_{24,1}^{r} & \varepsilon_{24,2}^{r} & \varepsilon_{24,3}^{r} & \ddots & \cdots & \cdots & \varepsilon_{24,n}^{r}\\ 0 & 0 & \cdots & \cdots & \ddots & \cdots & 0\\ \cdots & \cdots & \cdots & \cdots & \cdots & \ddots & \cdots \\ 0 & 0 & \cdots & \cdots & \cdots & \cdots & 0 \end{array}\right]\;\left[\begin{array}{c} y_{1}^{rs}\\ y_{2}^{rs}\\ y_{3}^{rs}\\ \cdots \\ \cdots \\ \cdots \\ y_{n}^{rs} \end{array}\right]$$

where *O^rs^* denotes the carbon emissions from the construction sector transferred from region *s* to region *r*, ε^*r*^ denotes the full carbon emission factor for the construction sector in region *r*, and *Y^rs^* denotes the portion of end‐use of goods and products from the sectors in region *r* for region *s* (carbon transfers are measured in tons).

### 2.2 Construction of a National Carbon Emission Transfer Network for the Construction Industry

The carbon emission transfer matrix of each province calculated above depicts the carbon transfer relationship among provinces in detail. However, the size of the carbon transfer coefficients and strength of the carbon transfer association among different nodes (provinces) differed. In general, the role of connecting edges with larger carbon transfers is more critical in the network, whereas connecting edges with smaller carbon transfers plays a negligible role in the network; therefore, the carbon emission transfer matrix was screened to construct the inter‐provincial carbon transfer network important for our study.

To determine critical values, most models use subjective empirical values, such as 0.1, 0.5, or average values. However, these methods have certain limitations owing to their subjectivity. We used the Weaver–Thomas index (later referred to as the W–T index) to determine the critical value in an endogenous manner. The screening process was as follows

1) Determine the critical values of the carbon emission transfer network matrix. Starting from the column vector of the carbon emission transfer matrix, the Weaver index was used to determine the *n* critical values corresponding to the *n* columns β_1_,β_2_,β_3_,…,β_*n*_.

2) Determine the carbon emission transfer correlation 0–1 matrix of China's construction industry *T*. Let *B*(*i*, *j*) be an element in the correlation coefficient matrix *B*, then

(7) $$T\left(i,j\right)=\left\{\begin{array}{c} B\left(i,j\right)\geq \beta_{n}\\ B\left(i,j\right)<\beta_{n} \end{array}\right.$$

*t_ij_* is an element of matrix *T*. If *t_ij_* = 1, then a strong correlation exists between provinces *i* and *j* . *t_ij_* = 0 indicates no strong correlation between provinces *i* and *j*.

3) The 0–1 matrix obtained in the previous step was used as the basis. In matrix *T*, *t_ij_* = 1 indicates an edge between provinces *i* and *j*, and vice versa; there is no edge between provinces *i* and *j* . Based on this, a carbon emission transfer network model for China's construction industry was established.

### 2.3 Data Sources

To facilitate data analysis and collation, this study considered factors such as data accessibility and objectivity. It combines the statistical data of China and its provinces to determine the scope of this study's research in 30 provinces, autonomous regions, and municipalities in China (excluding Tibet Autonomous Region, Hong Kong Special Administrative Region, Macao Special Administrative Region, and Taiwan Province). The provincial codes are listed in **Table** **3**. In addition, to maintain consistency with the energy consumption table sectors, we combined the original input–output table into 27 sectors. Because the latest year of the interregional input–output table is 2017, this study uses input–output samples as the interregional input–output table for the 10 years from 2007 to 2017. The data for the energy table for each year are from the China Energy Statistics Yearbook, the data for the material consumption table for the construction industry are from the China Construction Industry Statistics Yearbook, and the interregional input–output tables for the 30 provinces are from Liu Weidong's compilation for 2007^[^ ^34^ ^]^ and 2010^[^ ^35^ ^]^ and from the CEADs' compilation for 2012, 2015, and 2017.^[^ ^36^ ^]^

**Table 3:** Abbreviations of provinces.

Abbreviations of provinces	Provinces	Abbreviations of provinces	Provinces
BJ	Beijing	QH	Qinghai
TJ	Tianjin	NX	Ningxia
HE	Hebei	NM	Inner Mongolia
SX	Shanxi	LN	Liaoning
SH	Shanghai	JL	Jilin
JS	Jiangsu	HL	Heilongjiang
ZJ	Zhejiang	AH	Anhui
FJ	Fujian	SD	Shandong
JX	Jiangxi	SC	Sichuan
HA	Henan	SN	Shaanxi
HB	Hubei	GS	Gansu
GX	Guangxi	XJ	Xinjiang
HI	Hainan	GD	Guangdong
CQ	Chongqing	YN	Yunnan
GZ	Guizhou	HN	Hunan

## 3 Measurement of Interregional Carbon Emission Transfers

### 3.1 Carbon Emissions from Construction by Province

#### 3.1.1 Characteristics of Temporal Tends in Carbon Emissions from the Construction Sector by Province

**Figure 1:**
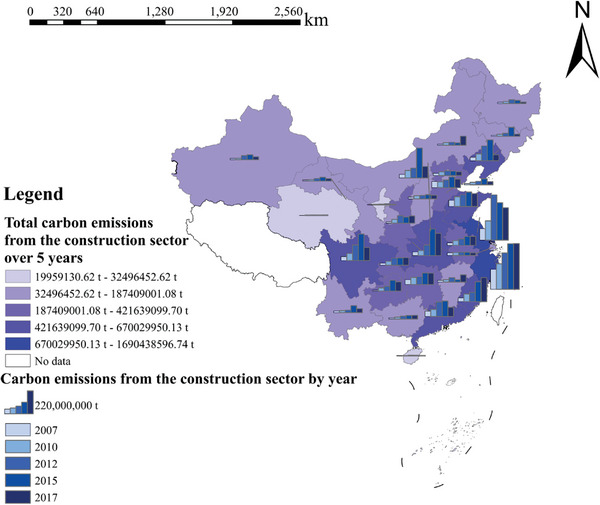
Total carbon emissions from the construction sector during the study period and carbon emissions from the construction sector by year.

According to the identified sources of carbon emissions from the construction industry in China from 2007 to 2017, carbon emissions were measured in each province of the construction industry, and the results of the calculation using the ArcGIS software are graphically displayed in **Figure** **1**.

Figure 1 shows that carbon emissions from the construction industry in most provinces in China in 2017 are greater than those from the construction industry in 2007, which can be divided into three categories. First, the carbon emissions from the construction industry have been in an upward trend from 2007 to 2017, including in Zhejiang, Guangxi, Fujian, Hunan, Anhui, and Chongqing. In the second case, carbon emissions from the construction industry first increased and then decreased between 2007 and 2017, including in Heilongjiang, Hebei, Beijing, Shandong, Hainan, Jiangsu, Shanghai. Third, there was a decline from one year during 2007–2017, and the remaining years, including in Xinjiang, Gansu, Ningxia, Yunnan, Guizhou, Sichuan, Guangdong, Henan, Jilin, Liaoning, Shanxi, and Tianjin, showed an upward trend. Over the 5‐year period, Zhejiang, Jiangsu, Shandong, Sichuan, Guangdong, and Henan had the greatest overall carbon emissions, as these provinces are economically developed and populous and have a strong demand for construction. Ningxia, Gansu, Qinghai, Guangxi, and Guizhou lag behind, as most of these provinces are located in the western region, have poor economic strength and a high net outflow of population in recent years, and do not have a strong demand for the construction industry.

In addition, combining the geographic location and the main carbon sources influencing the carbon emissions from the construction industry in each province shows that the main carbon sources influencing the carbon emissions from the provinces geographically closer to each other are similar.

#### 3.1.2 Characteristics of the Evolution of Carbon Emissions from the Construction Industry by Province

**Figure 2:**
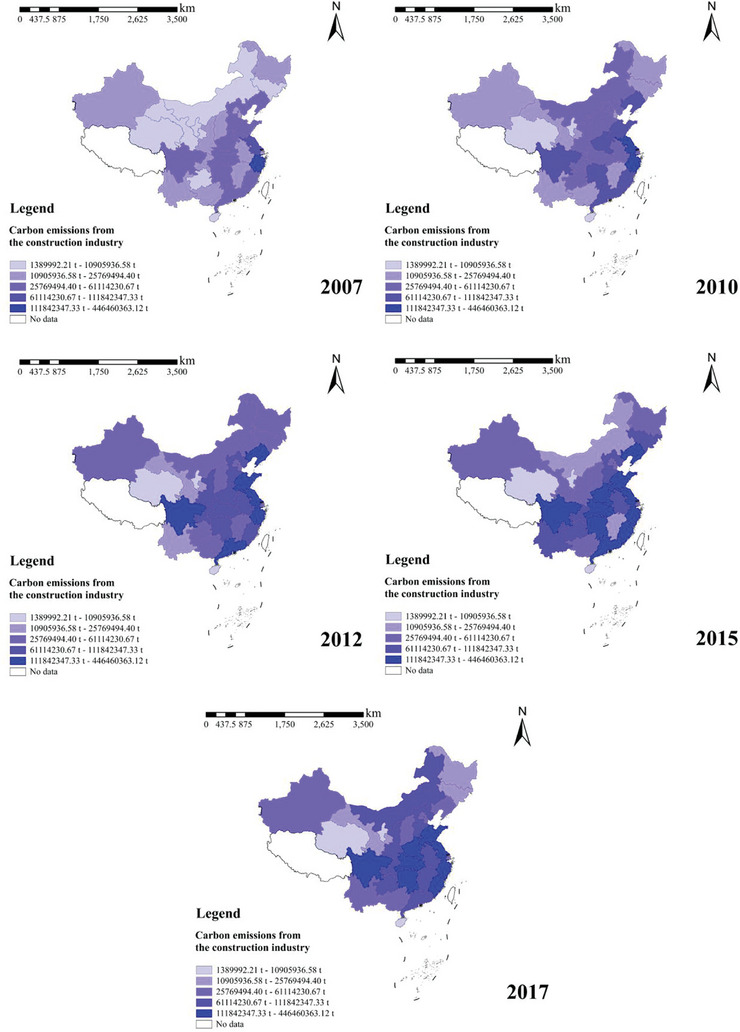
Carbon Emissions from Construction by Province in China (2007–2017).

To further understand the temporal and spatial distribution of carbon emissions from the construction industry in each province during 2007–2017 in China, ArcGIS software was utilized to draw vector maps of carbon emissions from the construction industry in each province for 2007, 2010, 2012, 2015, and 2017, and the results are shown in **Figure** **2**.

Figure 2 shows the temporal distribution of carbon emissions from the construction industry in each province for 2007, 2010, 2012, 2015, and 2017. The figure shows that China's carbon emissions from the construction industry from 2007 to 2017 were larger overall and showed a gradual increase in the trend (97 100 209 779 797.5 t in 2007 and 2 719 279 586 t in 2017), the number of provinces with carbon emissions from the construction industry above 50 million tons has increased significantly (5 provinces in 2007, 17 provinces in 2017), and the province with the most minor carbon emissions from the construction industry during the study period also had 1 389 992 t (Qinghai in 2007).

Analyzing Figure 2 shows that regions with larger carbon emissions from the construction industry are mainly distributed in the central and eastern regions of China, including the Hubei, Hunan, Shandong, Jiangsu, Zhejiang, Fujian, Guangdong, and Sichuan provinces. According to the National Bureau of Statistics, the gross output of the construction industry completed by construction enterprises in the Eastern region accounted for 51.7% of the national share in 2018. The central region accounted for 24.3%, which brought rapid urbanization and real estate investment to the eastern and central regions and increased carbon emissions. Provinces with low carbon emissions from the construction industry are mainly concentrated in the western and northeastern regions, including Qinghai, Ningxia, Xinjiang, Gansu, Inner Mongolia, Heilongjiang, Jilin, and Hainan, mainly because the economic development of these regions lags behind that of the eastern and central regions. Due to its low level of economic growth, the western region has limited fiscal revenue, resulting in a relative lack of investment in infrastructure construction. To a certain extent, this has restricted the development of the construction industry, as many large‐scale construction projects cannot be implemented in the western region due to a lack of sufficient financial support.

Second, infrastructure development in the western region has been relatively slow. Although the development of the western area in recent years has promoted the construction and improvement of infrastructure, a significant gap remains in the construction of transport networks, communication facilities, and other infrastructure in the western region compared to the eastern region. This lack of infrastructure affects the transport of building materials and the improvement of construction conditions and limits the development of the construction industry.

Finally, the industrial aggregation effect in the western region was not apparent. The development of the construction industry often requires the support of related industrial chains and ancillary services, and the western region lags behind in developing these aspects. Owing to the lack of sufficient industrial agglomeration, many construction‐related enterprises prefer to establish bases in the eastern and central regions with better infrastructure and greater market demand, further reducing construction activities in the western region.

### 3.2 Major Flows of Carbon Emission Transfers from the Construction Sector by Province

**Figure 3:**
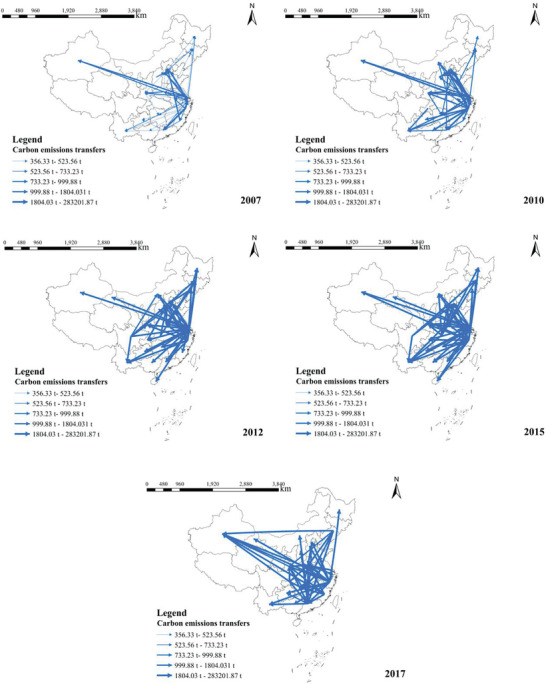
Main flows of carbon emission transfers from the construction sector, 2007–2017 Note: The carbon transfer measurements in the figure do not include carbon transfers from the provinces' own end‐use.

To further understand the direction of outflows from these provinces, we selected the top 50 flows of carbon emission outflows from the construction industry in terms of transfers from each province in each year for 2007 to 2017 (accounting for 51.46%, 53.66%, 71.36%, 82.75%, and 59.10% of outflows among provinces in the country in each year), and used the ArcGIS software to map the main flow direction of carbon emission transfer in the construction industry, as shown in **Figure** **3**.

The direction of the arrow points to the endpoint (i.e., the carbon emissions that should have been borne by region *r* are transferred to region *r* because of the final demand from region *s* to region *r*), and the thickness of the arrow indicates the size of the transfer.

As shown in the above figure, carbon emissions from the construction industry are mainly transferred to the eastern and central regions of China. From the time trend, the arrows become thicker with the year, that is, the amount of carbon transferred shows a year‐on‐year growth trend. Specifically, in 2007 and 2010, carbon transfers were concentrated in the eastern region, and after 2010, more central provinces also began to transfer carbon; however, carbon transfers have basically been centered in the eastern region, with a large amount of carbon transfers occurring outward.

In summary, except for 2017, China's carbon emission outflow direction was mainly concentrated in Jiangsu, Zhejiang, Shanghai, and the southern region, whereas the carbon emission inflow direction was primarily concentrated in Beijing, Tianjin, Hebei, and the northern region. Carbon transfer occurred frequently between the south and the provinces in 2017. Carbon transfer is not active in the northwestern and northeastern regions. The main reasons include the low level of economic development, the relatively small scale of the construction industry; land resource constraints, the limited growth of the construction industry due to land resources, the Northwest geographic conditions are complex, and the Northeast land resources are affected by the temperature and the relatively scarce impact of the agriculture. In addition, as inter‐provincial trade in the construction industry and its upstream and downstream sectors makes it easier to achieve economic cooperation between neighboring provincial administrative regions, carbon emission transfer paths in the construction industry also exist and are mainly within each economic region, with apparent geographical neighborhood effects.

## 4 Building and Analyzing a National Carbon Transfer Network for the Construction Industry

### 4.1 Construction of National Carbon Emission Transfer Network for the Construction Industry

Close interregional trade flows drive the flow of products and services, while also bringing about the transfer of large quantities of carbon dioxide emissions, gradually forming a complex multilateral relationship and a trade carbon emissions transfer network. A Complex Network refers to a network with self‐organization, self‐similarity, an attractor, a small world, and no scale in some or all properties. This study draws on the carbon emission transfer matrix from 2007 to 2017 based on the filtered carbon emission transfer matrix of the W–T index in Section 2. Subsequently, the position of each province in the carbon emission transfer network of the construction industry was quantified using the relevant indicators of the complex network theory.

#### 4.1.1 Node Degree

The nodal degree is an important index that represents the direction of carbon transfer and the strength of individual nodes in the carbon transfer network. In the carbon transfer network, the number of edges of node *i* that directly point to other nodes is called the out‐degree of node *i*, denoted by *Ok_i_*; and the number of edges of other nodes that directly point to node *i* is called the in‐degree of node *i*, denoted by *Ik_i_*, where the degree of node *i* is the sum of the node out‐ and in‐degree. Let the adjacency matrix of a carbon transfer network with *n* nodes be *A*  = (*a_ij_*)_*n* × *n*_ . Then

(8) $$Ok_{i}=\sum_{j=1}^{n}a_{ij}$$

(9) $$Ik_{i}=\sum_{i=1}^{n}a_{ji}$$

#### 4.1.2 Closeness Centrality

Closeness centrality considers the proximity of a particular node to other nodes in a network using the distance method. The Closeness centrality of node *i* is

(10) $$BC_{i}=\frac{N}{\sum_{j=1}^{N}d_{ij}},i\neq j$$

where *N* is the total number of nodes in the network and *d_ij_* is the distance between nodes *i* and node *j* as defined below

(11) $$d_{ij}=\frac{1}{w_{ik}}+\frac{1}{w_{kj}},k\neq i\neq j$$

#### 4.1.3 Eigenvector Centrality

Eigenvector centrality is a metric that reflects the importance of a node based on the degree of importance of its neighboring nodes. It is based on the idea that the importance of a node depends on the number of adjacent nodes and their significance of its adjacent nodes. The eigenvector centrality is calculated as follows

(12) $$EC_{i}=\lambda^{-1}\sum_{j\in N_{i}}a_{ij}EC_{j}$$

where *EC_i_* is the eigenvector centrality of province *i*, λ is a constant, *N_i_* is the number of nodes in the network, and *a_ij_* is the amount of carbon emission transfer from province *i* to province *j* generated by the construction industry.

### 4.2 Realization of National Carbon Transfer Network for the Construction Industry

Using the method in Section 2, 810 carbon transfers between provinces and regions were obtained. The coefficients of industry self‐association were removed, and 194, 225, 46, 88, and 205 strong associations between provinces and regions were screened using the Weaver index for 2007, 2010, 2012, 2015, and 2017, respectively. The interregional carbon emissions transfer network of China's construction industry from 2007 to 2017 was constructed using provinces as nodes and strong associations as connecting‐edge criteria.

**Figure 4:**
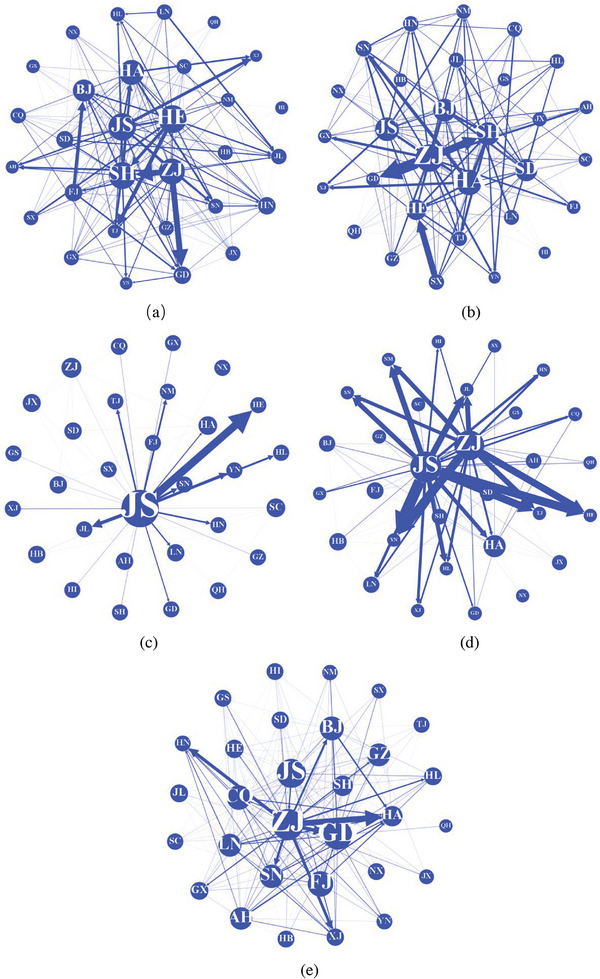
National Carbon Transfer Network for the Construction Sector, 2007–2017.

**Figure** **4**a–e shows the national carbon emission transfer networks of the construction industry in 2007, 2010, 2012, 2015, and 2017. The circles represent the provinces. The size of each circle represents the node degree; the more significant the circle, the larger the node degree. The directed edges represent the direction of the embodied carbon emissions transfer. The thickness of the edges represents the embodied carbon emissions from the construction industry in the province to other provinces. The thicker the edge, the larger the embodied carbon transfer.

### 4.3 Analysis of the National Carbon Transfer Network in the Construction Industry

Based on the realization of the national carbon transfer network for the construction industry, this study further analyzes the carbon transfer network for the construction industry through indicators related to the overall characteristics of the network, such as network density, indicators associated with the overall characteristics of the network, such as network density, and indicators associated with the individual characteristics of the network, such as closeness centrality and eigenvector centrality.

#### 4.3.1 Overall Network Characterization

Gephi's calculation function was used to understand the overall characteristics of the national carbon transfer network in the construction industry. Network density and other related indicators of the national carbon transfer network in the construction industry were measured from 2007 to 2017; the results are presented in **Table** **4**.

**Table 4:** Construction carbon transfer network overall indicators 2007–2017.

indicators	2007	2010	2012	2015	2017
Node numbers	30	30	30	30	30
Edge numbers	194	225	46	88	205
Density	0.223	0.221	0.061	0.112	0.236
Cluster coefficients	0.598	0.548	0.238	0.777	0.465
Average path lengths	1.924	1.718	1.898	2.059	1.551

The density of China's construction industry carbon emissions transfer network during 2007–2017 shows a trend of first decreasing and then increasing. The network density reflects the completeness of the network, and the more edges in the network, the denser the network is represented. This shows that from 2007 to 2017, the carbon transfer network of China's construction industry first became dense, then sparse, and then dense again; in other words, during this period, China's carbon emissions from the construction industry first became frequent, then sparse, and then frequent. China has been advocating green and low‐carbon development and the use of various types of clean energy in recent years. After 2015, China's rapid economic development, the demand for the construction industry has greatly increased, and domestic trade has become increasingly frequent, thus leading to more frequent carbon transfers in the construction industry and a greater network density. In addition, except for 2012 and 2015, the changes were relatively insignificant.

The average clustering coefficient of the carbon emission transfer network of China's construction industry during 2007–2017 shows a trend of first decreasing, then increasing, and finally decreasing. The clustering coefficient reflects the degree to which nodes are clustered. This indicates that China's construction industry's carbon emission transfer network first loosened from 2007 to 2012, became compact in 2015, and then loosened again in 2017. This suggests that the nodes (provinces) in China's construction carbon transfer network have undergone a process of change over the study period and that the position of each province in the carbon transfer network has not been static across years, but has been changing, with aggregation indicating that construction carbon transfers are more likely to occur in neighboring provinces and dispersal indicating that construction carbon transfers are more likely to take place in more distant provinces.

The maximum value of the clustering coefficient during the study period was 0.777, which indicates a 77.7% possibility that one province's construction carbon emissions transfer object in the network was a construction carbon emissions transfer object from other provinces. The entire network is strongly clustered.

The average path length of China's construction industry carbon emission transfer network during 2007–2017 shows a trend of decreasing, then increasing, and then decreasing again, with a smaller value indicating a closer connection between nodes in the network. During 2007–2017, the average path lengths were above 1.5. This means that any two provinces in the network can, on average, realize the transfer of carbon emissions from the construction industry through 1.5 other provinces. Therefore, in China's carbon transfer network for the construction industry, each province typically only needs to route through 1.5 provinces to realize the transfer of carbon emissions, which indicates that carbon emissions from China's construction industry generally do not occur in a long chain of transfers, and most occur between two or three provinces, Therefore, controlling carbon emissions from one of them plays an important role in controlling the entire carbon transfer network, particularly the provinces at the center of the carbon transfer pathway pointing to the center.

China's inter‐provincial construction carbon transfer network has a more significant average aggregation factor and shorter average path length. The network is characterized as a small world, and fluctuations in any node may affect other nodes and significantly impact the entire network.

#### 4.3.2 Individual Network Characterization

To further understand the individual characteristics of the national carbon transfer network in the construction industry, the degree, proximity, and eigenvector centralities of the national carbon transfer network in the construction industry from 2007 to 2017 were measured using Gephi's statistical calculation function. Spatial and temporal distribution maps of the network characteristics were plotted using the ArcGIS software.

##### Node Degree

The in‐degree and out‐degree are essential indicators of the node relationship, and directly reflect the relationship between carbon inflows and outflows between regions. When a region exports carbon to other areas, the greater the number of areas to which carbon flows, the greater the out‐degree, and the greater the number of other areas that export carbon to a region, the greater the in‐degree. Out‐degree and in‐degree reflect the breadth of the scope of carbon outflows and inflows from a network node, and directly indicate the number of industries with which carbon transfers occur. Suppose carbon reduction measures are implemented in regions with large in‐degrees and out‐degrees. In this case, their impact radiates to neighboring areas, resulting in a broader range of carbon reduction effects.

**Figure 5:**
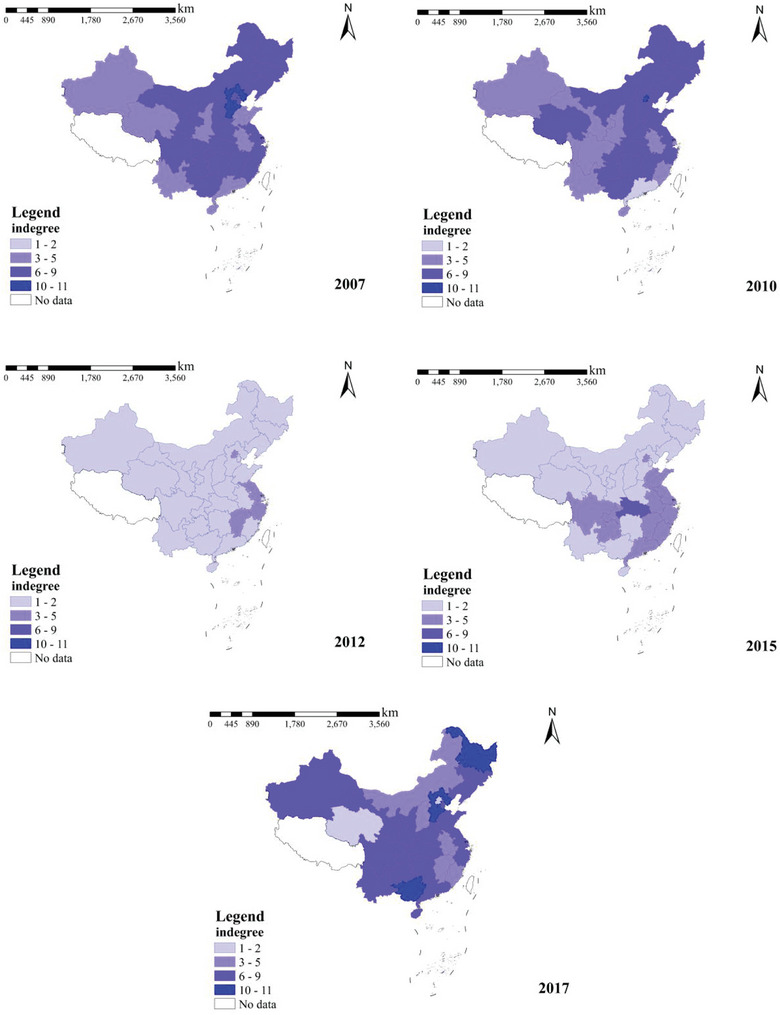
Temporal and spatial distribution of in‐degree, 2007–2017.

**Figure 6:**
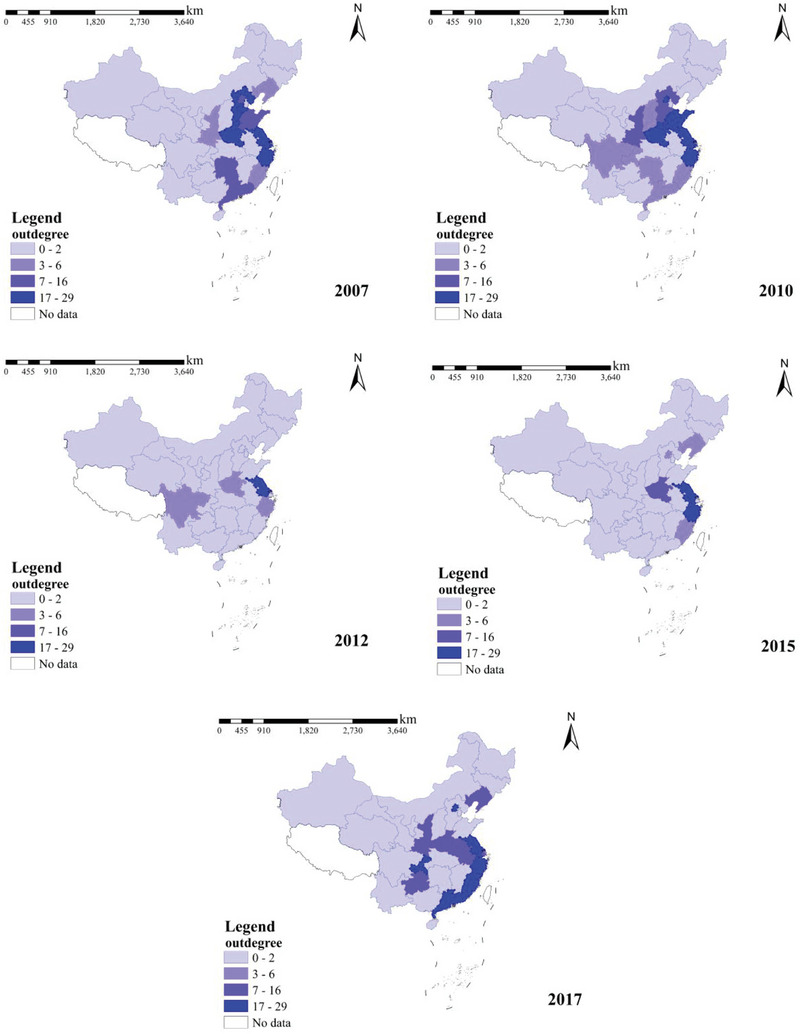
Temporal and spatial distribution of out‐degree, 2007–2017.

The spatial and temporal distributions of the in‐degree and out‐degree of the interregional carbon transfer network in China's construction industry are shown in **Figures** **5** and **6**.

The in‐degree reflects the construction industry's carbon inflow relationship in the carbon transfer network and expresses the amount of carbon transfer from one province to another; the larger the in‐degree, the more carbon transfer occurs in that province, and if carbon emissions are strictly regulated and controlled from the perspective of carbon inflow, it will significantly impact carbon emission reduction in that province. Based on this, the critical carbon inflow regions can be directly determined.

From 2007 to 2017, from the perspective of time trends, the in‐degree of most provinces showed a decreasing and then increasing trend, particularly in 2012 and 2015, when the in‐degree was concentrated in provinces, such as Jiangsu, Zhejiang, and Shanghai; that is, the endpoints of the carbon transfer were focused on these regions. Analyzed year by year, the total number of provinces with in‐degrees below three was 1, 2, 26, 23, and 3. This means that the construction industry in other provinces did not, or rarely, emit carbon during the study period. The total number of provinces with five or more in‐degrees was 26, 24, 1, 5, and 24. This indicates that the construction industries in other provinces often transferred carbon to these regions during the study period. The provinces with the highest in‐degree scores were Hebei, Beijing, Jiangsu, Hubei, and Hebei. Controlling the inflow of carbon emissions from these provinces will significantly impact carbon reduction from China's construction industry. Hebei and Beijing had two and one of the highest in‐degrees during this period, mainly because in 2007, the Beijing‐Tianjin‐Hebei Metropolitan Area Regional Plan was almost complete, depicting a blueprint for the development of the area with Beijing, Tianjin, and the Binhai New Area as the main lines of development. In 2014, the Chinese Premier, Li Keqiang, introduced a program to integrate Beijing, Tianjin, and Hebei in his government work report. Both these measures have led to the rapid development of the construction industry in the Beijing‐Tianjin‐Hebei region, which is supported in all aspects by many construction projects in other provinces.

The out‐degree can reflect the construction industry's carbon outflow relationship in the carbon transfer network and expresses the amount of carbon transfer from one province to other provinces; the larger the out‐degree, the more carbon transfer occurs in that province. If carbon emissions are strictly regulated and controlled from the perspective of carbon emission outflow, it will have a significant effect on carbon emission reduction in that province. Based on this, the critical carbon outflow regions can be determined directly.

From 2007 to 2017, the out‐degree of most provinces shows a decreasing and then an increasing trend, particularly in 2012 and 2015, in which the out‐degree is concentrated in provinces such as Jiangsu and Zhejiang; that is, the starting point of the carbon transfer is focused on these regions. In terms of out‐degree and in‐degree, Zhejiang and Jiangsu played essential roles in the national carbon transfer network of the construction industry. The year‐by‐year analysis shows that the total number of provinces with an out‐degree of less than two is 18, 16, 26, 24, and 18. This implies that the construction industry in these regions rarely emitted carbon to other provinces during the study period. The provinces with an out‐degree of more than 19 were 5, 4, 1, and 2. There were 5, 4, 1, 2, and 5 provinces with an out‐degree of 19 or higher. This indicated that the construction industry in these regions developed rapidly and frequently transferred carbon to other provinces during the study period. The out‐degrees of the remaining provinces ranged from 2 to 18. In the statistics of provinces without‐degrees of 19 or more during these 5 years, Jiangsu Province appeared 5 times. Zhejiang Province appeared 4 times, which indicates that these two provinces play an essential role in carbon transfer because Jiangsu and Shanghai are in China's Yangtze River Delta Economic Zone, which is the most substantial economic center of China's comprehensive strength, an important international gateway of the Asia‐Pacific, a globally crucial advanced manufacturing base, and the first to be among the world‐class urban agglomerations in the region. Zhejiang and Jiangsu have led the development of the construction industry, generating more carbon transfers with other provinces.

##### Closeness Centrality

**Figure 7:**
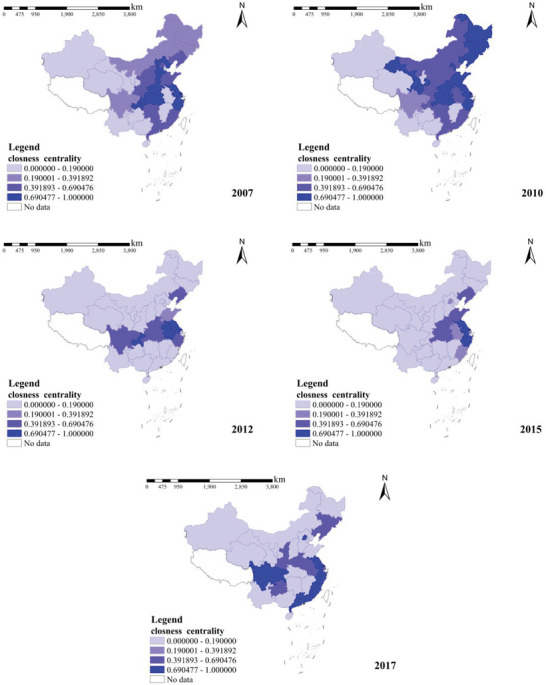
Temporal and spatial distribution of closeness centrality 2007–2017.

Closeness centrality mainly describes the total distance between provinces: for a node, the closer it is to other nodes, the greater its proximity centrality in the national carbon transfer network. A province with high proximity centrality means that it is close to other provinces in the carbon transfer, and that it is often with neighboring provinces in carbon transfer with geographic limitations. For such a province, controlling the carbon emissions of its neighboring provinces will result significantly decrease the province's carbon emissions. The spatial and temporal distributions of the closeness centrality of the interregional carbon transfer network in China's construction industry are shown in **Figure** **7**.

From the perspective of temporal trends, most provinces showed a trend of rising, then falling, and then rising again based on closeness. Centrality was higher in the central region in 2007, spread to most of the northern region in 2010, concentrated in the Yangtze River Basin in 2012; concentrated in Jiangsu, Zhejiang, Shanghai, Henan, Shandong, and other provinces in 2015; and concentrated in the coastal region in 2017. It has substantial spatial and temporal evolution.

The top five provinces in terms of proximity centrality in 2007 were Chongqing, Shanghai, Jiangsu, Hebei, and Zhejiang; those in 2010 were Tianjin, Zhejiang, Heilongjiang, Jilin, and Liaoning; those in 2012 were Jiangsu, Anhui, Chongqing, Henan, and Sichuan; those in 2015 were Jiangsu, Zhejiang, Henan, Liaoning, and Hubei; and those in 2017 were Sichuan, Zhejiang, Guangdong, Jiangsu, and Fujian. This indicates that these provinces are at the core of the network and are more likely to transfer carbon emissions from other provinces with a higher efficiency of carbon emission transfer. For this part of the province, the carbon emissions and carbon transfer of its neighboring provinces should be controlled, and the carbon transfer factor of this part of the province will be significantly improved after the carbon emissions of the neighboring provinces are controlled. These provinces can be roughly divided into three categories: first, the core provinces of the region, such as Sichuan, the core of the Southwest region; Liaoning, the core of the Northeast region; and Henan, the core of the Central region; second, resource‐rich provinces, such as Heilongjiang and Jilin; and third, economically strong provinces, such as Chongqing, Shanghai, Jiangsu, Zhejiang, Tianjin, and Guangdong. In addition, except for Sichuan (Southwest Regional Centre) and Gansu (rich in mineral resources), most of these provinces are located in the central and eastern regions of China and have close trade with other provinces in the construction industry. If these nodal provinces are further controlled in terms of formulating energy‐saving and emission‐reduction policies, better carbon emission reduction can be achieved.

##### Eigenvector Centrality

**Figure 8:**
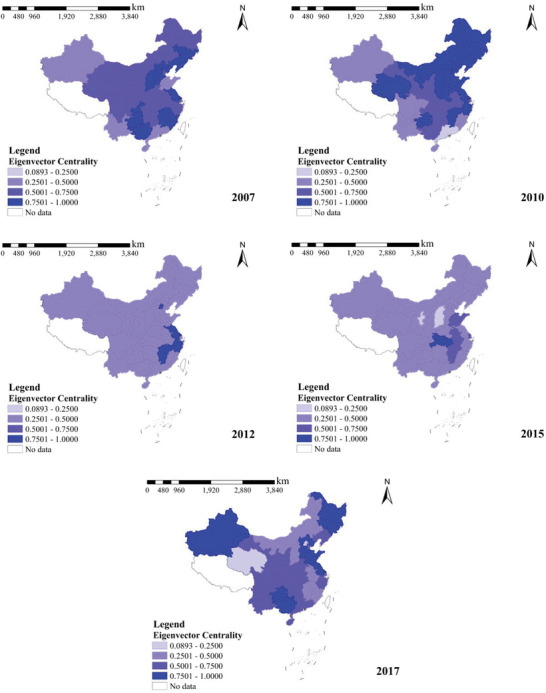
Temporal and spatial distribution of eigenvector centrality, 2007–2017.

A province is considered vital if it has more connected provinces, and the province connected to it is in a position of importance. This province was also regarded as necessary. The eigenvector centrality can be used to describe this indirect effect. In the construction industry's regional carbon transfer network, the more carbon transfer connections a province has with other provinces and the more the province it is connected to is in the center of the network, the more substantial the eigenvector centrality of that province. This indicates that provinces with higher eigenvector centrality have more provinces associated with them, and most of them are at the center of the carbon transfer network. In the carbon transfer network, provinces with higher eigenvector centrality typically have a better effect on carbon emission reduction in the entire carbon transfer network; therefore, more stringent control or environmental protection measures should be taken for these provinces. The spatial and temporal distributions of the centrality of the eigenvectors of the interregional carbon transfer network in China's construction industry are shown in **Figure** **8**.

From the temporal trend, the centrality of eigenvectors in most of the provinces shows a tendency to rise, then fall, and then rise again, from being scattered throughout the country in 2007 to concentrated in the northern region in 2010, to focus in provinces such as Beijing, Zhejiang, and Jiangsu in 2012, to concentrate in provinces such as Jiangxi, Anhui, Hubei, and Shandong in 2015, and then scatter throughout the country in 2017, which has a solid spatial and temporal evolution.

In terms of each year, the top five provinces in terms of eigenvector centrality in 2007 were Chongqing, Beijing, Jiangsu, Hebei, and Fujian; those in 2010 were Beijing, Jilin, Tianjin, Liaoning, and Qinghai; those in 2012 were Jiangxi, Zhejiang, Jiangsu, Beijing, and Anhui; those in 2015 were Hubei, Shanghai, Jiangxi, Shandong, and Anhui; and those in 2017 were Hebei, Guangxi, Heilongjiang, Guizhou, and Jilin. This indicates that these provinces strongly correlate with the key provinces in the network and that their indirect influence is more substantial. Owing to the strong carbon emission impact on other provinces in the network, controlling these provinces will have a favorable effect on the overall carbon emission reduction of the carbon transfer network.

Shanghai, Jiangsu, Zhejiang, Anhui, Jiangxi, Hubei, and Fujian are located in the middle and lower reaches of China's Yangtze River Basin economic belt, with developed economies and complete manufacturing and service industries, attracting a large amount of labor and resource inputs, and thus having a high eigenvector centrality.

As the three provinces in the Beijing‐Tianjin‐Hebei national metropolitan area, Beijing, Hebei, and Tianjin have been tilted by a series of national policies in recent years, particularly the construction of the Xiong’ a New Area, which provides new ideas and methods for the future development and revitalization of new areas in China.

Chongqing, Guangxi, and Guizhou, the core provinces in the southwest region, have transformed from moats into roads in recent years, and numerous challenging engineering projects have been inaugurated in Guizhou and Guangxi. Such engineering projects often require a large amount of resource investment, and thus have high eigenvector centrality.

Heilongjiang, Jilin, and Liaoning are three provinces in Northeast China. They are rich in natural resources and Liao‐Zhong‐Nan is one of China's four major industrial bases. Their resource advantages support eigenvector centrality in the network.

Shandong, as the province with the second‐largest population, third‐largest GDP, and a complete range of industries, is at the forefront of the country in terms of economic strength and labor resources. This provides the necessary conditions for developing a construction industry with high eigenvector centrality.

## 5 Conclusions and Policy Recommendations

From the spatial distribution of carbon emissions and the characteristics of the national carbon transfer network in the construction industry, the following conclusions were drawn.

1. From the viewpoint of China's carbon emissions from the construction industry, most provinces remain in an upward trend, which reduced in 2017 and is closely related to China's advocacy for green buildings and environmentally friendly materials in recent years. In addition, most regions with high carbon emissions are concentrated in the eastern coastal area. In contrast, Hubei and Henan in the central region emitted more carbon, and only one province in the western region, Sichuan, had high carbon emissions.
2. Regarding the carbon transfer networks of China's construction industry, Hebei, Jiangsu, and Beijing were the provinces with the largest in‐degrees during the study period. The provinces with the largest out‐degrees were Jiangsu and Zhejiang, indicating that these provinces often transfer carbon to other provinces, or other provinces often transfer carbon to them in the carbon transfer network. The provinces with high closeness centrality were mainly classified into three categories: first, the core provinces of the region, such as Sichuan, the core of the southwest region; Liaoning, the core of the northeast region; and Henan, the core of the central region; second, the resource‐rich provinces, such as Heilongjiang and Jilin; and third, the economically strong provinces, such as Chongqing, Shanghai, Jiangsu, Zhejiang, Tianjin, and Guangdong. Provinces with higher eigenvector centrality are similar to those with higher closeness centrality, most of which are national core strategic and resource‐rich regions.

Based on the above research, this study proposes the following suggestions:

1. Promote the development of green buildings, and support and promote green building standards. Starting with the aspects of building design, material selection, construction, maintenance, and use; reducing the energy consumption and environmental impact of buildings; promoting the recycling of waste, wastewater, and low‐carbon building materials; upgrading building construction equipment and construction technology; realizing the organic integration of lifelong and long‐term carbon reduction; and pushing forward the “dual‐carbon” target process.
2. Improve the program to determine the total carbon emissions and allocate responsibility to provincial areas. China's provinces with more carbon transfers should actively respond to the national call to assume specific carbon‐trading responsibilities. Jiangsu, Shanghai, Zhejiang, Beijing, Hebei, Shandong, Tianjin, Hubei, Sichuan, and other economically developed provinces have more carbon transfers to Xinjiang, Inner Mongolia, Yunnan, Liaoning, Jilin, Heilongjiang, and other economically less developed regions; the former transfers large quantities of carbon emissions to the latter to satisfy the needs of the local industry. The former has transferred a large amount of carbon emissions to the latter to meet local industrial needs; therefore, the former should give the latter carbon emission reduction technology, capital, and other aspects of assistance to compensate for this part of the transfer.
3. Formulate regionally differentiated carbon emissions reduction policies and peak carbon targets. China has a vast territory, and there are significant differences between provinces and regions in terms of the level of development of the construction industry, economic strength, production technology, endowment of resource factors, and division of labor in the domestic value chain. Provinces on the eastern and southern coasts, Beijing, and Tianjin have a solid economic foundation, a higher gross value of construction output, and an advanced level of technology. They should optimize the selection of construction materials, use innovative technologies and methods to reduce energy consumption, and accelerate the green development of the construction industry to consolidate its carbon emission reduction, minimize the transfer of carbon from the construction industry, strive to take the lead in reaching a peak, and steadily push forward carbon neutrality. The underdeveloped construction industry in the Central and Western regions should adhere to green and low‐carbon development, strengthen the use of clean energy to meet the demand for local and external transfer, and control the increase in carbon emissions. Energy saving and carbon reduction should be the primary goals for the West, Northeast, and other resource‐rich regions. They should also strengthen the development of clean energy, reduce the economic growth of the construction industry based on the dependence on high‐carbon sectors, gradually realize the decoupling of economic growth and carbon emission growth, and strive for regional peak carbon nodes in tandem with national synchronization.

## Conflict of Interest

The authors declare no conflict of interest.

## Data Availability

The data that support the findings of this study are available from the corresponding author upon reasonable request.
